# Identification of persistent benthic assemblages in areas with different temperature variability patterns through broad-scale mapping

**DOI:** 10.1371/journal.pone.0177333

**Published:** 2017-05-10

**Authors:** N. David Bethoney, Liuzhi Zhao, Changsheng Chen, Kevin D. E. Stokesbury

**Affiliations:** School for Marine Science and Technology, University of Massachusetts Dartmouth, New Bedford, Massachusetts, United States of America; Universita degli Studi di Genova, ITALY

## Abstract

Ecosystem-based management is a place-based approach that considers the relationships between system parts. Due to the complexity of ecosystems in the marine environment it is often difficult to define these relationships in space and time. Maps illustrate spatial concepts. Here we promote ecosystem-based spatial thinking by layering datasets from a larger project that mapped benthic fauna, substrate characteristics, and oceanic conditions on monthly, annual and decadal time scales along the U.S. continental shelf. By combining maps of persistent benthic megafauna and bottom temperature variability over approximately 90,000 km^2^, we identified wide spread benthic animal assemblages and regional disparity in temperature variability. From a broad-scale perspective the locations of the assemblage appear to be related to sea scallop population dynamics and indicate potential regional differences in climate change resiliency. These findings offer information on a scale that correlates with marine spatial planning, and could be used as a starting point for further investigation. To spur additional analysis and facilitate their linkage to other datasets, these datasets are available through public, online data portals. Overall, this study demonstrates how the growth of maps from single to multiple elements can help promote and facilitate the multifactor, ecosystem-based thinking needed to support regional ocean planning.

## Introduction

Ecosystem-based fisheries management should enhance marine resource management [1]. This approach often requires complicated, interdisciplinary analysis appropriately interpreted, synthesized, and communicated to policy-makers and stakeholders. This complexity often limits implementation to the conceptual stage [1, 2]. Maps can synthesize data across disciplines and help people understand spatial concepts. Pauly et al. [3] states, “Ecosystem-based implies that fisheries-relevant ecological processes, and fisheries themselves, need to be documented in the form of maps”. A stepwise process of mapping and layering habitat and animal datasets will improve communication and understanding of ecosystem-based fisheries management [2]. Maps and datasets of individual animal distributions or environmental features are an important first step as they promote the understanding of these components and document their initial conditions [2]. They also facilitate the aggregation of system parts, which are needed for analysis of biodiversity and animal-environment interactions, and can be combined with spatial information about human activities to aid in marine spatial planning.

The need for a more spatially explicit, comprehensive picture of the marine environment and human activities is exemplified along the U.S. Northeast Shelf. In this region, federal, state, tribal and fishery management council members, with stakeholder engagement, have developed the Northeast Ocean Plan to enhance the management and development of the region’s ten primary ocean resources. The plan summarizes a process, within existing regulatory frameworks, to achieve goals for each of these resources in an ecosystem-based approach [4]. A key component of the plan is an online, public data portal that provides access to data related to each primary ocean resource to aid in developing a multifaceted, regional perspective on ocean management issues.

The Northeast Ocean Plan identified six overarching research priorities for future datatypes to be added to or generated from the data portals. In collaboration with The Nature Conservancy, we conducted a regional mapping project to create data products that addressed research priorities 1. “Improved understanding of marine life and habitats” and 5. “Characterize changing conditions and resulting impacts to existing resources and uses” within the Northeast Ocean Plan [4]. Specifically, the goals were to create maps that could synthesize the distribution of benthic animals, description of the seafloor habitat, and fluctuations in ocean conditions. The project leveraged data from an image based sea scallop (*Placopecten magellanicus*) survey [5] and an oceanographic model [6] to produce maps and datasets that provided baseline knowledge of 12 benthic megafauna groups, 5 substrate characteristics, and 6 physical variables on monthly, annual and decadal time scales. The spatial resolution of these products was defined by the New England Fishery Management Council’s Swept Area Seabed Impact (SASI) model grid. The SASI model is a quantitative tool for evaluating fisheries management alternatives by examining the tradeoffs between habitat impacts and fishery yields [7]. All maps and datasets from this project are available through the Northeast and Mid-Atlantic data portals and the Nature Conservancy’s Conservation Gateway.

Here the overall methods and data products of the project are described and a sub-set of the data is used to produce a map identifying persistent benthic megafauna assemblages in relation to bottom temperature variability, to demonstrate how visual layering can help promote ecosystem-based, spatial thinking. Abundance data was used to map areas of persistent concentrations of benthic megafauna and the maps were layered to identify the composition and location of benthic megafauna assemblages. The assemblage map was then combined with a decadal bottom temperature variability map. The result was a spatially explicit identification of animal assemblages and the temperature variability they persisted in from 2003 to 2012 over approximately 90,000 km^2^ of the U.S. Northeast Shelf.

## Methods

**Fig 1:**
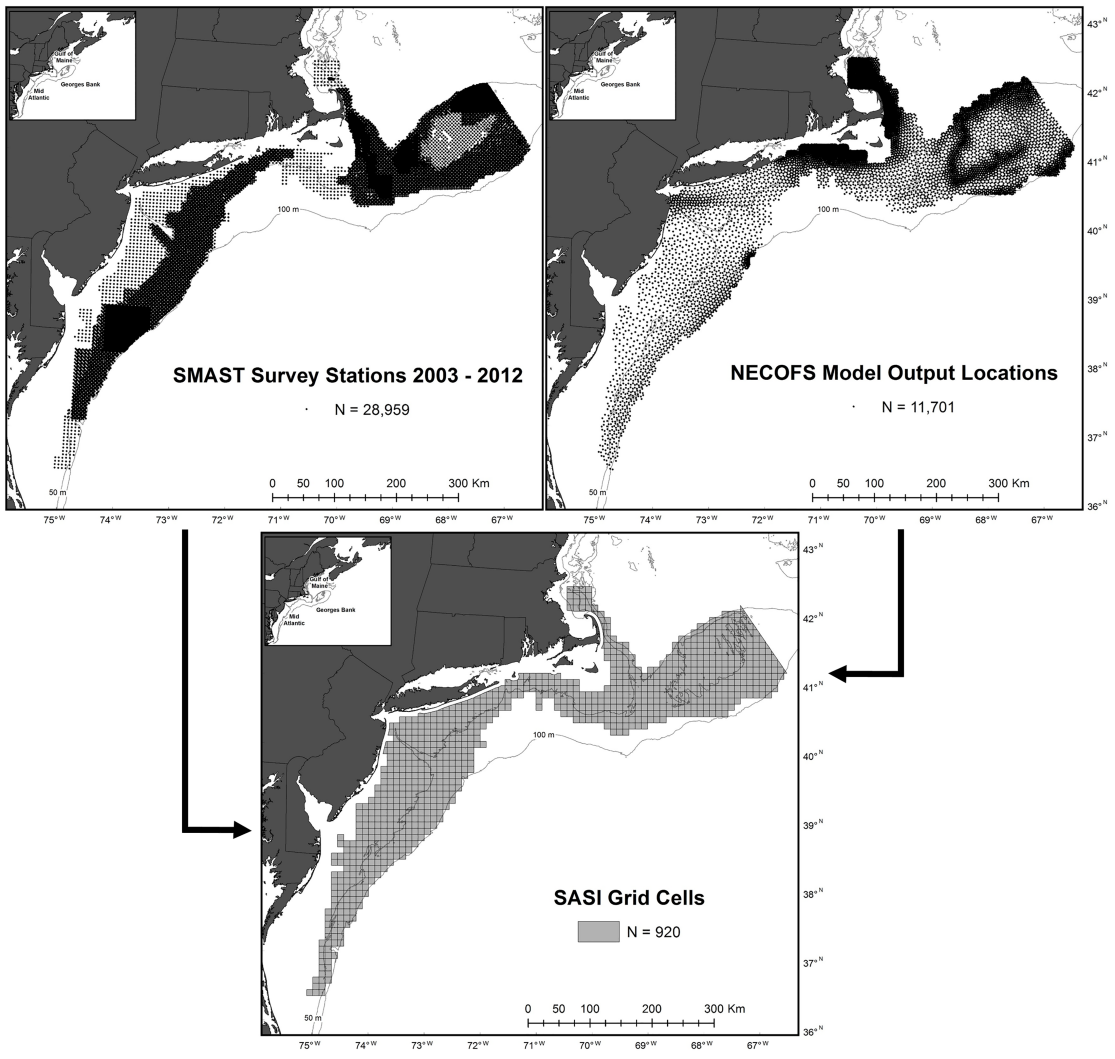
The spatial extent of datasets integrated into Swept Area Seabed Impact (SASI) model grid. The University of Massachusetts Dartmouth School of Marine Science and Technology (SMAST) broadscale drop camera survey was conducted from 2003–2012 on a 5.6 km grid, with finer scale surveys on 1 to 4 km grids in certain years, to monitor sea scallop populations. The Northeast Coastal Ocean Forecast System (NECOFS) was used to hindcast oceanographic conditions.

With the aid of the commercial sea scallop fishing industry, the University of Massachusetts Dartmouth, School of Marine Science and Technology (SMAST) surveyed the U.S. Northeast Shelf from the southern Mid-Atlantic to the USA-Canadian border on eastern Georges Bank from 2003 to 2012 between late April and the end of June (Fig 1). The survey used underwater video footage and a two-stage sampling design with stations sampled on regular grids and four quadrats sampled at each station [5]. At each station a pyramid was lowered to the seafloor. Mounted on the pyramid were two downward facing cameras that provided 2.84 m^2^ and 0.60 m^2^ quadrat images of the seafloor. Another camera, mounted parallel to the seafloor, provided a side profile of the quadrat area to aid in species identification. The vessel was allowed to drift approximately 50 m and the pyramid was lowered to the seafloor again to obtain a second quadrat; this was repeated four times. Sampling four quadrats at each station increased the sampled area to 11.36 m^2^. Within each quadrat, macroinvertebrates and fish were counted and the substrate was identified. When possible macroinvertebrates and fish were identified to species, otherwise animals were grouped into categories based on taxonomic orders [8]. Sediment types observed in quadrat images were mapped over the spatial extend of the survey using the methods of [9]. No permission for this sampling was required as the method was non-invasive and did not involve endangered or protected species. However, scientific research letters of acknowledgment were obtained from the National Oceanic and Atmospheric Administration for each survey to coordinate research activities with enforcement of federal regulations.

Monthly and annual hindcasts of bottom and surface temperature (°C) and salinity, and average and maximum bottom stress magnitude (N m^-2^) generated by the Northeast Coastal Ocean Forecast System (NECOFS) were used to characterize environmental variables in the benthic survey area. The oceanic components of the NECOFS model were created based on the Finite-Volume Coastal Ocean Model [6]. This model has assimilated sea surface temperature and sea surface height on a daily basis and monthly temperature and salinity measurements since 1978. Hindcast fields have been validated by comparing results with many in-situ measurements of tidal water level change, temperature and salinity throughout the water column, and surface currents [6, 10–12].

**Fig 2:**
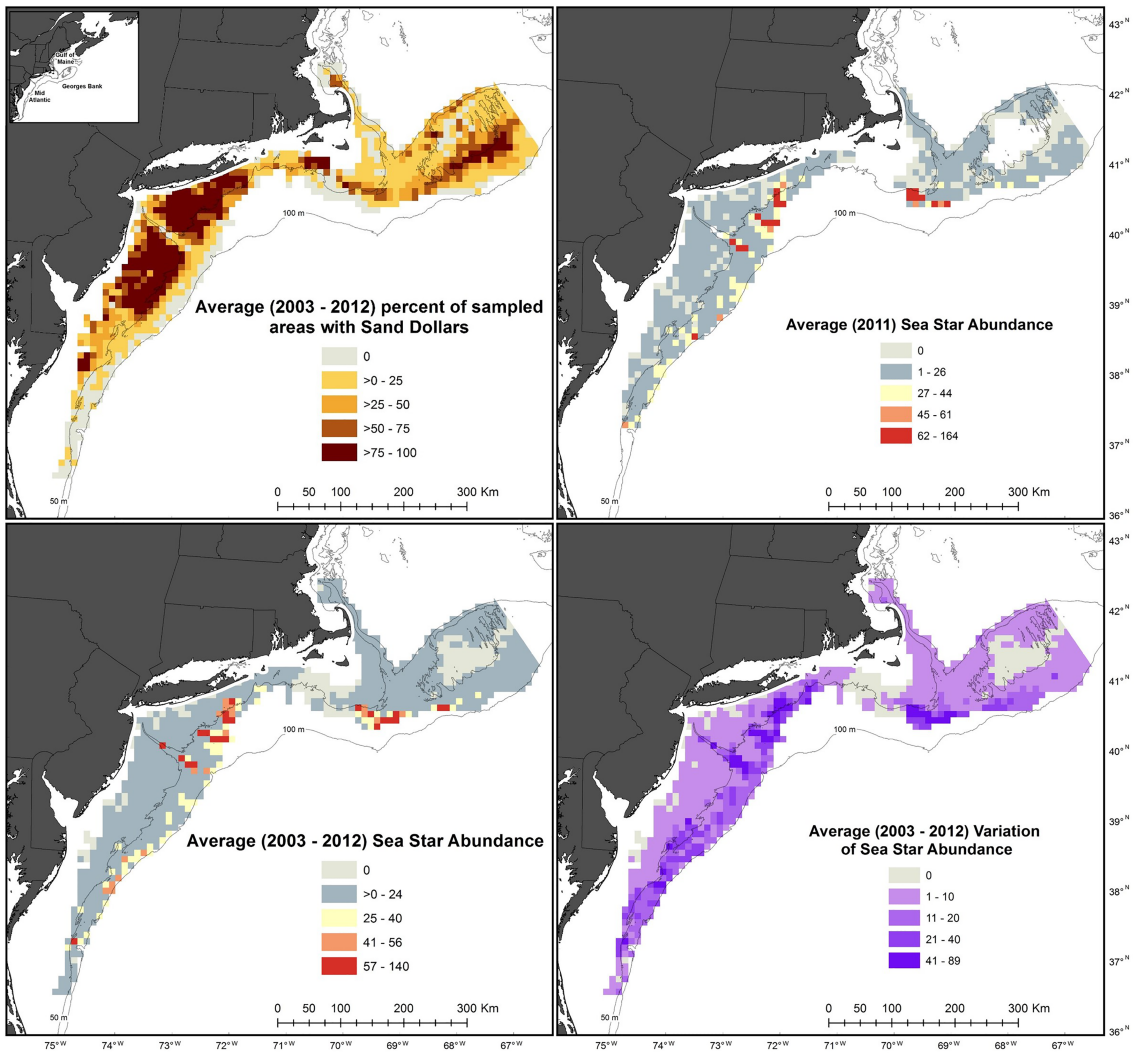
Visualization of benthic animal dataset created from integration of The University of Massachusetts Dartmouth School of Marine Science and Technology drop camera survey data into the Swept Area Seabed Impact model grid. The dataset includes abundance information for eight animal groups (sea stars, sea scallops, hermit crabs, skates, red hake, moon snails, crabs, and flatfishes) as well as presence/absence data for four additional groups (bryozoa/hydrozoa, sand dollars, sponges, and burrowing species) on an annual and decadal scale. In addition, each uniquely identified cell contains information on the number, average depth, and variation between the survey stations used to create the data within the cell.

The Swept Area Seabed Impact grid consists of approximately 6,200 uniquely identified cells, a core of about 5,600 10 x 10 km^2^ cells and about 600 irregularly shaped cells along the border with areas less than 100 m^2^, in U.S. federally managed waters from Maine to North Carolina. Benthic survey stations from 2003–2012 were overlaid onto the grid and only grid cells with a survey station within 5.6 km of each border were utilized to prevent overextrapolation of the benthic survey data [13]. This resulted in a domain of 920 grid cells(Fig 1) and data products that match the spatial scale utilized by regional managers [7]. Several survey stations were outside of, but within 5.6 km of grid cell and were assigned to the closest cell. Animal observations from each station within or closest to a grid cell were then averaged to create annual and decadal abundance values for each cell. Substrate information from the benthic survey was already incorporated into the SASI model, so it was not repeated here [7]. The analysis was limited to the 12 most frequently observed animal groups in the largest camera view, consisting of count or presence/absence data, to focus map and data products on the dominant benthic megafauna (Table 1). For each grid cell integrated benthic survey information included average depth, average animal count (abundance or number of quadrats per a station present), and the standard deviation of animal counts from the survey stations assigned to the cell for each year from 2003 through 2012 and for all 10-years combined (Fig 2).

**Table 1:** The 12 most frequently observed animal groups, in order of most to least observed, in the drop camera survey dataset.

Animal Group
Sea Stars
Sea Scallops (*Placopecten magellanicus*)
Bryozoans/Hydrozoans
Sand Dollars (*Echinarachnius parma*)
Hermit Crabs
Skates
Sponges
Red Hake (*Urophycis chuss*)
Moon snails (*Euspira heros*)
Crabs
Flatfishes
Holes (burrowing animals)

Bryozoa/Hydrozoa, sand dollars, sponges, and holes were only recoded as present or absent. Animal groups without scientific names, indicate animals grouped into categories based on taxonomic orders (refer to [8] for detailed information on the species that make up these groups).

**Fig 3:**
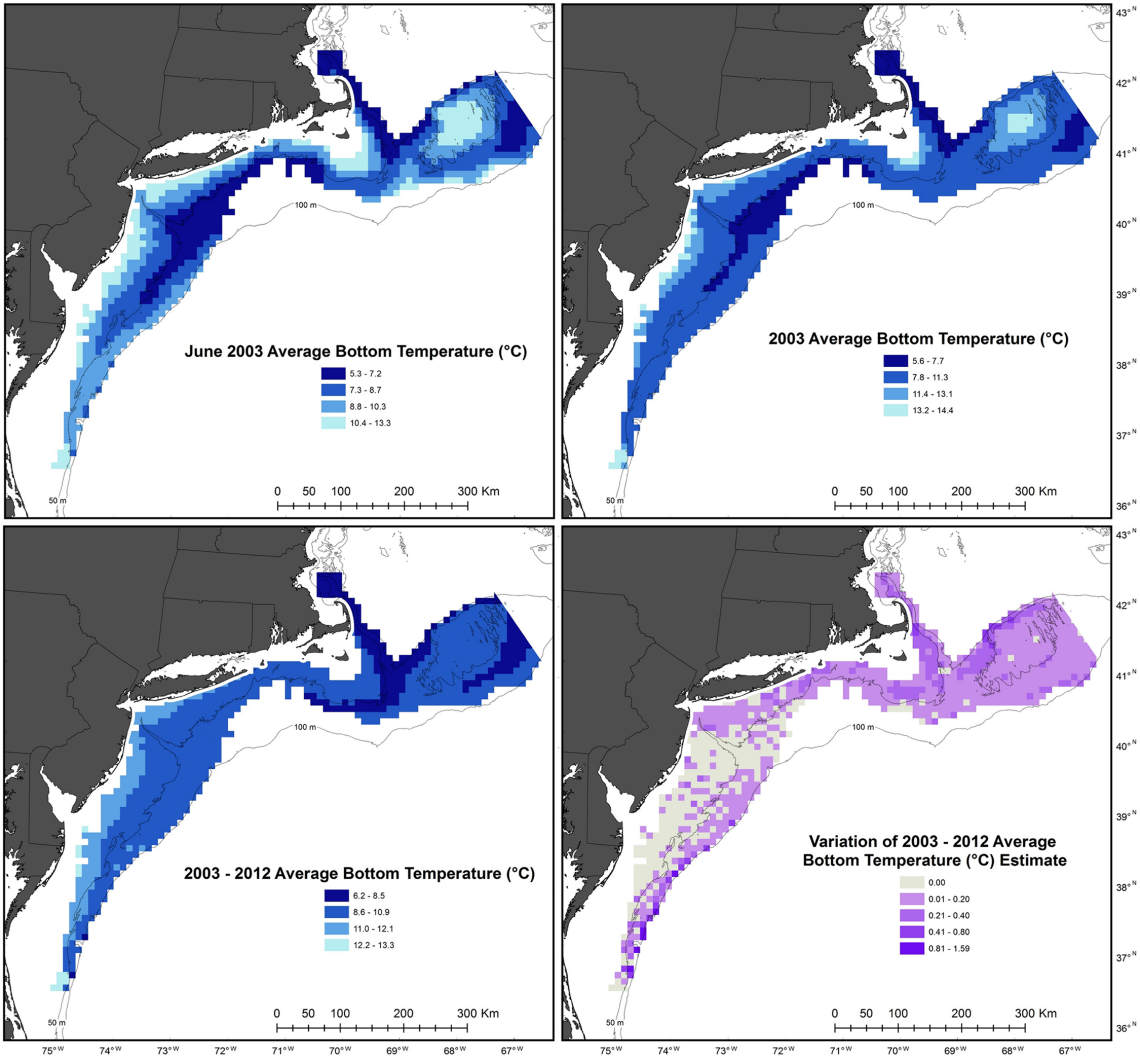
Visualization of environmental dataset created from integration of The Northeast Coastal Ocean Forecast System data into the Swept Area Seabed Impact model grid. The dataset includes information for six environmental variables (surface and bottom temperature and salinity, and maximum and average bottom stress) on monthly, annual and decadal scales. In addition, each uniquely identified cell contains information on the number and variation between the model output locations used to create the data within the cell.

Oceanographic hindcast data were integrated into the grid cells to create monthly, annual and decadal values of each environmental variable in a similar manner as the benthic survey data. However, NECOFS output locations are structured so that their density varies proportionally with the rate of depth change and environmental modeling difficulty. As a result, 24 grid cells did not have model output locations within them and were assigned the value from the closest output location. Environmental data consisted of average monthly, annual and decadal values, associated standard deviations, and the number of model output locations used to create these values (Fig 3). All spatial analysis was conducted using ArcGIS (version 10.2).

Areas of persistent, high concentrations for each animal group were mapped if a grid cell contained an abundance above the 50^th^ percentile within a year for more than half of observed years. Annual percentiles for Georges Bank and the Mid-Atlantic were calculated separately for cells observed for at least 6 years. The individual animal group maps were layered to identify cells with concentrations of multiple groups. Persistent benthic megafauna assemblages were defined as cells containing more than 3 concentrations of individual animal groups.

Areas with unusually high or low annual temperatures were also mapped. For each year, monthly and annual average bottom temperature values were subtracted from the 10-year average values by cell. The positive and negative residuals were averaged separately to identify the average higher and lower variation. These average variation values were increased by one standard deviation to identify the range of “normal” deviation from the 10-year average. Cells that contained residuals outside of this “normal” range were identified by year. Maps of these bottom temperature anomalies and persistent benthic megafauna assemblages were overlaid to display the degree of overlap. To test if a statistical correlation supported patterns revealed by mapping, a Spearman correlation was conducted to test for a relationship between the number of persistent, high animal concentrations and temperature anomalies per SASI grid cell. This correlation is a simple way to test for a relationship between two variables and therefore aligned with the exploratory nature of the manuscript [14]. A non-parametric test was chosen because the frequency distribution of both variables was skewed towards zero.

## Results

**Fig 4:**
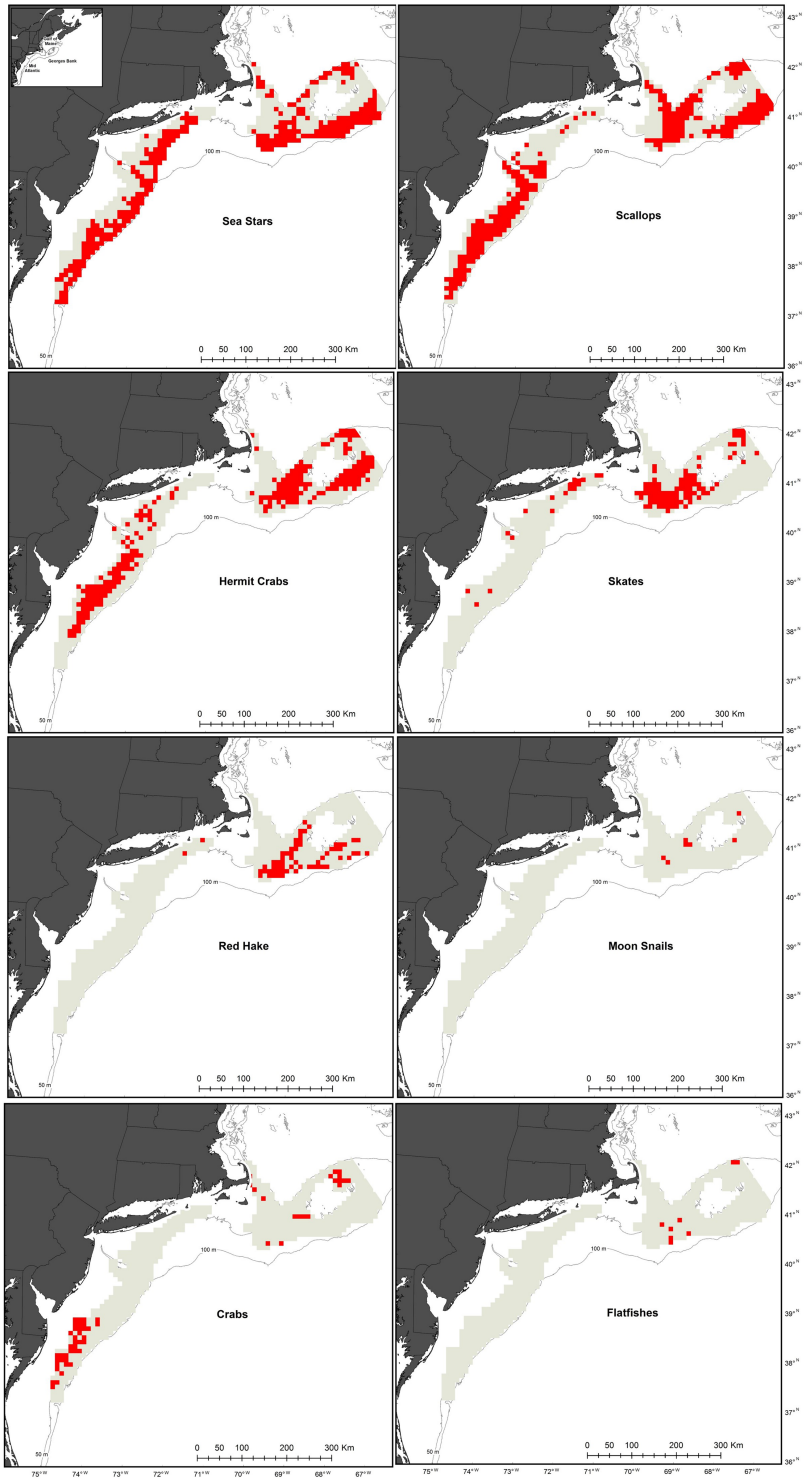
Areas of consistent concentrations of eight benthic animal taxa.

Sea stars, sea scallops, and hermit crabs were the dominant animal groups as concentrations were present over broad areas of both Georges Bank and the Mid-Atlantic (Fig 4). Skate concentrations were present in parts of northeastern Georges Bank, west of Georges Bank and the northern Mid-Atlantic (Fig 4). Crabs concentrations were primarily located in the nearshore, southern portion of the Mid-Atlantic, but also in some of the shallower areas of Georges Bank (Fig 4). Concentrations of red hake were primarily observed on Georges Bank. Moon snail and flatfish concentrations only occurred on Georges Bank, but were much less frequent than any other animal group (Fig 4).

**Fig 5:**
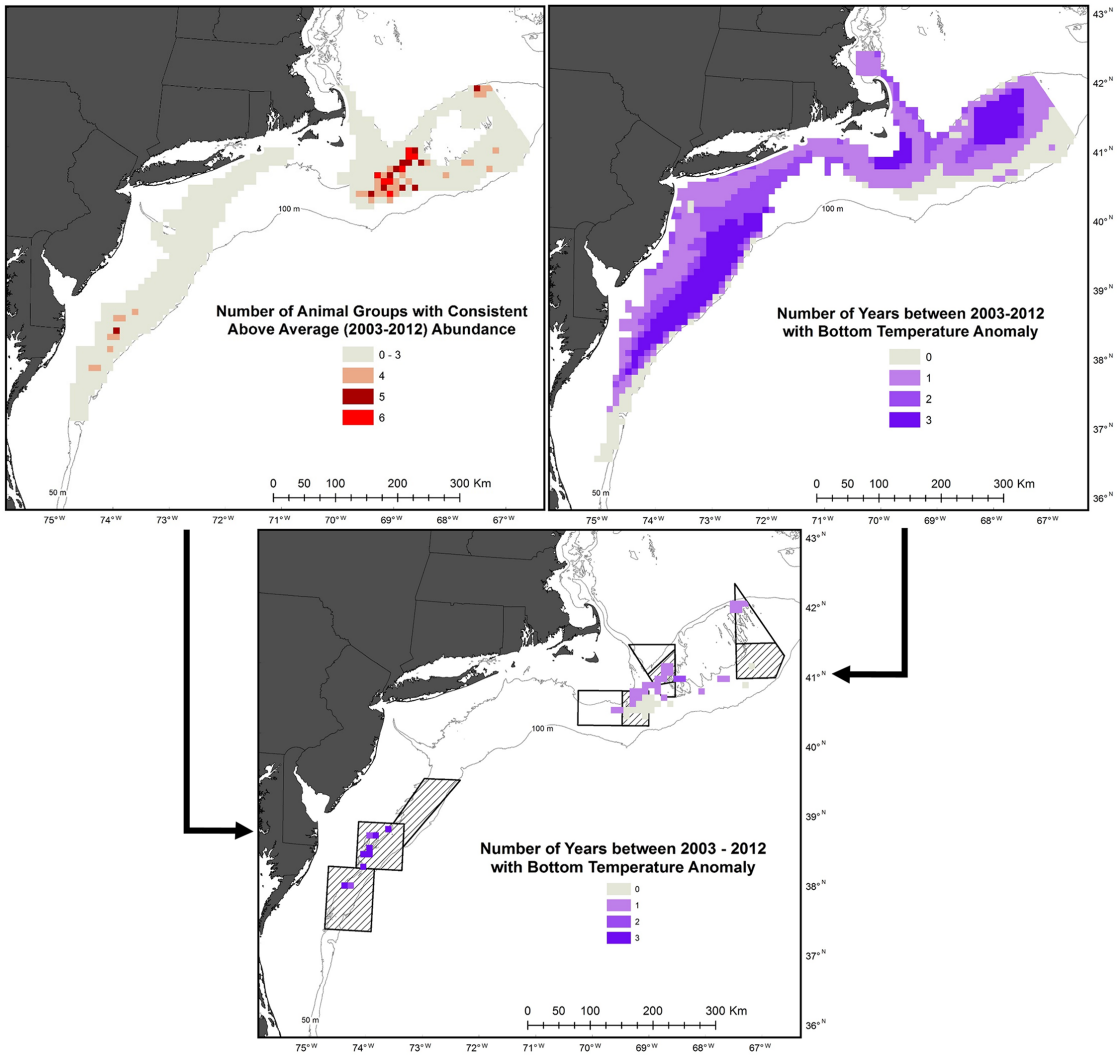
Areas and frequency of consistent benthic animal concentrations and bottom temperature anomalies. The bottom panel displays bottom temperature anomalies only for areas with persistent benthic assemblages. Outlined areas are closed to mobile, bottom fishing gear except for the hatched areas, which are periodically opened to sea scallop fishing.

There were three clusters of persistent benthic megafauna assemblages, two on Georges Bank and one in the southern Mid-Atlantic (Fig 5). The largest was a swath of 3,500 km^2^ (35 cells) in the southwestern region of Georges Bank (Fig 5). Within this swath, 3,000 km^2^ included concentrations of both sea stars and sea scallops. When four or five animal concentrations were present, the assemblage generally had a combination of hermit crabs, red hake, and skates or all three in addition to sea scallops or sea stars. However, in most of the southernmost area of the swath (900 km^2^) hermit crabs were absent. The 800 km^2^ with six animal groups consisted of sea stars, sea scallops, hermit crabs, red hake, and skates plus flatfishes or moon snails (with one exception). Moon snail concentrations were part of persistent benthic megafauna assemblages only when six animal groups were present. In contrast, crab concentrations were never part of persistent benthic megafauna assemblages when six animal groups were present. The assemblages in the 500 km^2^ area (5 cells) in the northeast portion of Georges Bank were comprised of concentrations of sea stars, sea scallops, hermit crabs, and skates except for one 100 km^2^ area. This area included concentrations of flatfishes, but not sea stars. Flatfish concentrations were also included in a 100 km^2^ area 5 animal group concentrations (Fig 5). The areas in the Mid-Atlantic, totaling 900 km^2^, were all comprised of concentrations of sea stars, sea scallops, hermit crabs, and crabs except for the northern most 100 km^2^. This area included concentrations of skates, but not sea stars. Skate concentrations were also included in the one 100 km^2^ area with 5 animal group concentrations (Fig 5).

**Fig 6:**
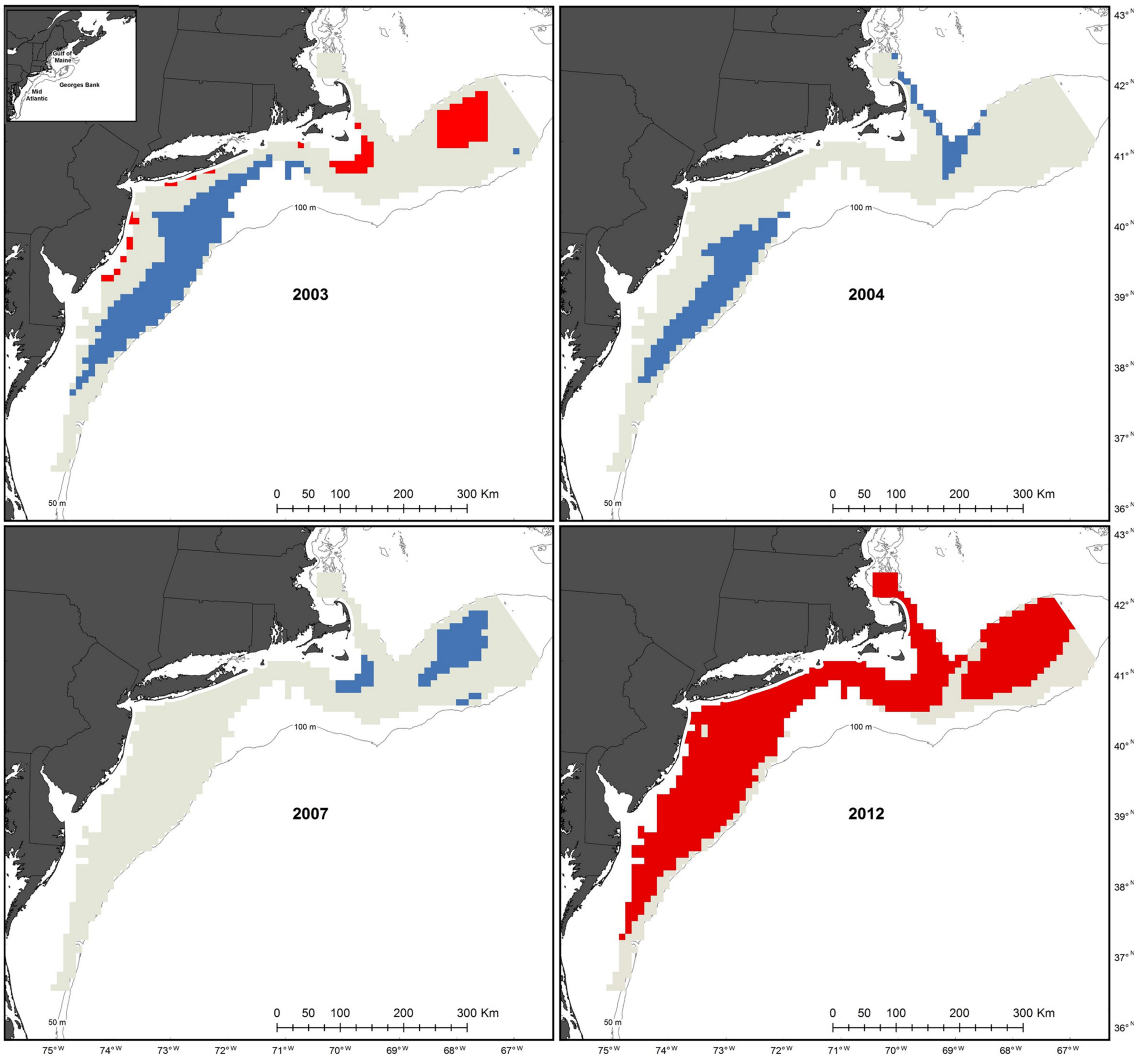
Areas of bottom temperature anomalies. An anomaly was present when an annual temperature value was one standard deviation higher (red) or lower (blue) than the average variation from 2003–2012. Only years from 2003–2012 with temperature variations outside of this range are displayed.

Unusually warm or cold average bottom temperature occurred during at least one year over 76,500 km^2^ of the 90,000 km^2^ examined (Fig 5). Average bottom temperatures in 2012, which resulted in mean temperatures 1.6 to 2.2°C higher than the 10-year average, impacted almost the entire region (Fig 6). The deeper areas of Georges Bank and the deeper or southern most areas of the Mid-Atlantic were the only large groupings of cells not affected by warm temperatures in 2012. Most of these areas had no anomalies in any other year (Fig 5). Areas with two years of unusual temperatures were affected by the warm temperatures in 2012 and cold temperatures (1.5 to 2.6°C lower than the 10-year average) in 2003 or 2004 (Fig 6). Three areas had three years of unusual bottom temperatures (Fig 5). The two in the Georges Bank area had abnormally low temperatures in 2003 and 2007, but abnormally high temperatures in 2012. Similarly, the group along the shelf break in the Mid-Atlantic had two years of anomalous cold average bottom temperature (2003 and 2004) and an abnormally high bottom temperature in 2012 (Fig 6).

Overlaying the animal assemblage and bottom temperature maps revealed assemblages in areas of temperature stability and variability. Most (98%) of the persistent assemblages on Georges Bank were found in areas with 1 or less years of anomalous temperature (Fig 5) and the number of animal concentrations was negatively correlated to the number of years of anomalous temperature (r_S_(319) = -0.36, p < 0.01). In contrast, all the persistent assemblages in the Mid-Atlantic were found in areas with 2 or 3 years of unusual temperatures (Fig 5) and the number of animal concentrations was positively correlated to the number of years of anomalous temperature (r_S_(321) = 0.40, p < 0.01).

## Discussion

The concentration maps represent decadal distribution snapshots of several prevailing taxonomic groups, which can be utilized individually or collectively. Individually, each concentration map is a spatial representation of the environment in which each taxonomic group persisted in, and can be interpreted as a habitat map [15]. Collectively, these maps identified persistent assemblages which are important for understanding benthic community structure. By identifying an assemblage, it becomes clearer which animals may be affected by positive or negative impacts to another member of the benthic community. For example, sea scallops, sea stars, and hermit crabs appear to form the foundation of a benthic community on both Georges Bank and in the Mid-Atlantic (Figs 4 and 5). These three taxa were present in 65% of assemblages on Georges Bank and 89% of assemblages in the Mid-Atlantic, despite the 56 different possible combinations of choosing three groups from eight. The result implies that a mechanism links these three taxa and that changes in abundance or distribution of one could impact the other two.

The mechanism linking sea scallops, sea stars, and hermit crabs may be related to areas of sea scallop concentrations with high mortality. Sea scallops are one of the dominant benthic macroinvertebrates on the continental shelf of the northwest Atlantic occurring from the Gulf of St. Lawrence to Cape Hatteras, North Carolina in a wide range of depths (2–384 m) and temperatures (1–20°C), but strongly associated with sand-gravel substrate [16]. They are ideally shaped for living in high flow conditions and create depressions in the sediment to improve feeding, which may create refuge for species less adapted to strong currents or that need surfaces to attach to [5, 16]. The predator-prey relationship between sea stars and sea scallops is well documented [17, 18], but the presence of hermit crabs in this group is new. Hermit crabs are scavengers that increase in abundance in areas after fishing [19]. The link may be especially strong to sea scallop fishing, due to the discarding of sea scallop guts at-sea [8]. When the assemblages are mapped in the context of areas open (periodically or continually) or closed to sea scallop fishing, 85% are within areas open to scallop fishing (Fig 5). However, hermit crabs were also present in all but one of the assemblages in areas that have been closed to fishing for approximately 20 years [20]. These areas and most of the areas open to fishing experienced extremely large sea scallop settlement events in the past fifteen years, which indicate areas of high abundance, but also high juvenile mortality [21]. Though these extremely large sea scallop settlement events are relatively uncommon, they may indicate areas consistently receiving sea scallop spat that can be preyed upon by sea stars and scavenged by hermit crabs. Lastly, large natural mortality events of sea scallops have been documented in areas corresponding with the southern swath on Georges Bank [22, 23]. From a broad-scale perspective, this assemblage appears linked to areas with a combination of sea scallop fishing, high sea scallop juvenile settlement, or substantial adult sea scallop die-offs.

The bottom temperature anomaly maps indicate different patterns of variability on Georges Bank and Mid-Atlantic. On Georges Bank, bottom temperatures fluctuated more in the shallow, center of the shelf, in contrast the Mid-Atlantic Bight tended to be more variable along the shelf break in (Fig 5). Besides 2012, when the entire study area was unusually warm, there was no synchrony between anomalies in the two different regions. For example, in 2003, the center of Georges Bank had an unusually high annual temperature, while the Mid-Atlantic shelf break had an anomalously cold annual temperature. This aligns with the general characterization of these areas as different sub-regions [24] and suggests the impacts of climate change may be different in these two regions, though they are often grouped together for large scale climate change analysis [25]. This is exemplified by the last glacial maximum, which covered Georges Bank, but left the Mid-Atlantic as a refuge area for marine taxa [26].

Combining the assemblage and temperature anomaly maps can help understand regional or species level differences in climate change resilience. The sea scallop-sea star-hermit crab assemblage was observed in portions of the Georges Bank without temperature anomalies, but also in the areas of the Mid-Atlantic that had the most bottom temperature variability in the study regions. This result could be viewed as a representative, snapshot of overall variability in the region and is supported by the wider range of annual bottom temperatures in the Mid-Atlantic compared to Georges Bank from 1977 to 2013 [27]. This highlights the importance of considering past environmental variability when investigating the resilience of these animals to long term environmental change as persistence in areas with annual fluctuations as high as 2.2°C above the decadal averages demonstrates resilience to extreme temperature conditions. Due to this, the assemblage in the Mid-Atlantic may have an improved level of resiliency to temperature change compared to the same group of animals on Georges Bank that adapted to a habitat with less temperature variability. For example, long-clawed hermit crab (*Pagurus longicarpus*) populations are genetically different between Massachusetts and the Carolinas due to isolation by distance since the last glacial maximum, suggesting physiological differences may exist as well [28]. Sea scallops also exhibit genetic evidence for adaption to regional environmental conditions, though sea scallop populations show connectivity between Georges Bank and the Mid-Atlantic [29]. If these assemblages represent one patchy population of each species without regional adaptations to environmental conditions, results suggest an overall resilience to temperature variability. In both regions, the distribution of the persistent assemblages may be driven by factors such optimal depth, water flow, or substrate type which may supersede the impact of changes in temperature variability [30]. The central portion of Georges Bank, with high temperature variability, was not surveyed enough to quantify persistence. However, low sea scallop abundance was observed in the years it was surveyed and its exclusion from the annual survey area was due to low utilization by the U.S. sea scallop fleet also suggesting low sea scallop abundance.

Mapping multiple ecosystem elements over a 90,000 km^2^ area yielded insight into two major research topics needed to better understand the U.S. northeast continental shelf. Each step created data products that were combined to show a wide spread benthic assemblage persisted in habitats with different temperature variation patterns. Understanding this initial state difference indicates potential differences in climate change resiliency and is an important first step needed to identify regime shift [31]. These findings and data products offer information on a scale that correlates with marine spatial planning, and should be used as starting point for further investigation. For example, more focused examination of the large areas with persistent assemblages may reveal a more refined distribution, especially since some of the grid cells include areas closed and open to fishing and assemblages appeared more diverse on Georges Bank than in the Mid-Atlantic. Additional environmental factors such as substrate type or bottom stress magnitude to characterize the environment around persistent assemblages or inclusion of more animal taxa would also build upon these initial findings. This type of approach could be executed by utilizing the data products from the larger project or other spatially specific information available on public data portals. Overall, ecosystem ecology grew from species ecology as the field evolved to place more emphasis on the interactions between different species, including humans [32]. The growth of maps from single to multiple elements echoes this evolution and can help promote and facilitate the multifactor, ecosystem-based thinking needed to support regional ocean planning.

## References

[1] Fogarty MJ. The art of ecosystem-based fishery management. Canadian Journal of Fisheries and Aquatic Sciences. 2014; 71:479–490.

[2] Cogan CB, Todd BJ, Lawton P, Noji TT. The role of marine habitat mapping in ecosystem-based management. ICES Journal of Marine Science. 2009; 66:2033–2042.

[3] Pauly D, Watson R, Christensen V. Ecological Geography as a Framework for a Transition Toward Responsible Fishing In: Sinclair M, Valdimarsson G, editors. Responsible Fisheries in the Marine Ecosystem. Wallingford: CAB International; 2003 pp. 87–102.

[4] Northeast Regional Planning Body. Northeast Ocean Plan. 2016. http://neoceanplanning.org/wp-content/uploads/2016/10/Northeast-Ocean-Plan_Full.pdf

[5] Stokesbury KDE, O’Keefe CE, Harris BP. Fisheries Sea Scallop, *Placopecten magellanicus* In: Shumway S, Parsons GJ, editors. Scallops: Biology, Ecology, Aquaculture, and Fisheries 3^rd^ ed Amsterdam: Elsevier B.V; 2016 pp. 719–736.

[6] Chen C, Haung H, Beardsley RC, Xu Q, Limeburner R, Cowles GW, et al Tidal dynamics in the Gulf of Maine and New England Shelf: An application of FVCOM. Journal of Geophysical Research. 2011; 116:C12010.

[7] New England Fisheries Management Council. Omnibus Essential Fish Habitat (EFH) Amendment 2 Draft Environmental Statement Appendix D: The Swept Area Seabed Impact (SASI) approach: a tool for analyzing the effects of fishing on Essential Fish Habitat. 2011. http://archive.nefmc.org/habitat/index.html

[8] Stokesbury KDE, Harris BP. Impact of limited short-term sea scallop fishery on epibenthic community of Georges Bank closed areas. Marine Ecology Progress Series. 2006; 307:85–100.

[9] Harris BP, Stokesbury KDE. The spatial structure of local surficial sediment characteristics on Georges Bank, USA. Continental Shelf Research 2010; 30:1840–1853.

[10] Beardsley RC, Chen C, Xu Q. Coastal flooding in Scituate (MA): A FVCOM study of the 27 December 2010 nor’easter. Journal of Geophysical Research: Oceans. 2013; 118:6030–6045.

[11] Sun Y, Chen C, Beardsley RC, Xu Q, Qi J, Lin H. Impact of current-wave interaction on storm surge simulation: A case study for Hurricane Bob. Journal of Geophysical Research: Oceans. 2013; 118:2685–2701.

[12] Li Y, Fratantoni PS, Chen C, Hare JA, Sun Y, Beardsley RC, et al Spatio-temporal patterns of stratification on the Northwest. Progress in Oceanography. 2015; 134:123–137.

[13] Adams CF, Harris BP, Stokesbury KDE. Geostatistical comparison of two independent video surveys of sea scallop abundance in the Elephant Trunk Closed Area, USA. ICES Journal of Marine Science. 2008; 65:995–1003.

[14] Hampton RE, Havel JE. Associations between Two Measurement Variables: Correlation In: Introductory Biological Statistics. Long Grove: Waveland Press Inc.; 2014 pp. 129–138.

[15] Brown CJ, Smith SJ, Lawton P, Anderson JT. Benthic habitat mapping: A review of progress towards improved understanding of the spatial ecology of the seafloor using acoustic techniques. Estuarine, Coastal and Shelf Science. 2011; 92:502–520.

[16] Hart DR, Chute AS. Essential Fish Habitat Source Document: Sea scallop, *Placopecten magellanicus*, life history and habitat characteristics, 2nd ed NOAA Tech. Memo 2004; NMFS-NE-189.

[17] Hart DR. Effects of sea stars and crabs on sea scallop *Placopecten magellanicus* recruitment in the Mid-Atlantic Bight (USA). Marine Ecology Progress Series 2006; 306:209–221.

[18] Marino MC II, Juanes F, Stokesbury KDE. Spatio-temporal variations of sea star *Asterias* spp. distributions between sea scallop *Placopecten magellanicus* beds on Georges Bank. Marine Ecology Progress Series. 2009; 382:59–68.

[19] Collie JS, Escanero GA, Valentine PC. Effects of bottom fishing on the benthic megafauna of Georges Bank. Marine Ecology Progress Series. 1997; 155:159–172

[20] Murawski SA, Brown R, Lai HL, Rago PJ, Hendrickson L. Large-scale closed areas as a fishery-management tool in temperate marine ecosystems: the Georges Bank experience. Bulletin of Marine Science. 2000; 66:775–798.

[21] Bethoney ND, Asci S, Stokesbury KDE. Implications of extremely high recruitment events into the US sea scallop fishery. Marine Ecology Progress Series. 2016; 547:137–147

[22] Stokesbury KDE, Harris BP, Marino MC II, Nogueira JI. Sea Scallop Mass Mortality in a Marine Protected Area. Marine Ecology Progress Series. 2007; 349; 151–158.

[23] Levesque MM, Inglis SD, Shumway SE, Stokesbury KDE. Mortality assessment of Atlantic sea scallops (*Placopecten magellanicus*) from gray-meat disease. Journal of Shellfish Research. 2016; 35(2):295–305.

[24] Link J, Overholtz W, O’Reilly J, Green J, Dow D, Palka D, et al The Northeast U.S. continental shelf Energy Modeling and Analysis exercise (EMAX): Ecological network and model development and basic ecosystem metrics. Journal of Marine Systems. 2008; 74:453–474.

[25] Hare JA, Morrison WE, Nelson MW, Stachura MM, Teeters EJ, Griffis RB, et al A Vulnerability Assessment of Fish and Invertebrates to Climate Change on the Northeast U.S. Continental Shelf. PLOS ONE. 2016; 11(2):e0146756 10.1371/journal.pone.0146756 26839967 PMC4739546

[26] Maggs CA, Castilho R, Foltz D, Henzler C, Taimour-Jolly M, Kelly J, et al Evaluating signatures of glacial refugia for north Atlantic benthic marine taxa. Ecology. 2008; 89(11):108–22. 10.1890/08-0257.1 19097488

[27] Kleisner KM, Fogarty MJ, McGee S, Barnett A, Fratantoni P, Greene J, et al The effects of sub-regional climate velocity on the distribution and spatial extent of marine species assemblages. PlOS ONE. 2016; 11(2):e0149220 10.1371/journal.pone.0149220 26901435 PMC4762943

[28] Young AM, Torres C, Mack JE, Cunningham CW. Morphological and genetic evidence for variance and refugium in Atlantic and Gulf of Mexico populations of the hermit crab *pagurus longicarpus*. Marine Biology. 2002; 140:1059–1066.

[29] O’Leary JK, Micheli F, Airoldi L, Boch C, De Leo G, Elahi E, et al The Resilience of Marine Ecosystems to Climatic Disturbances. BioScience. 2017; 67(3):208–220.

[30] Van Wyngaarden M, Snelgrove PVR, DiBacco C, Hamilton LC, Rodríguez-Ezpeleta N, Jeffery NW, et al Identifying patterns of dispersal, connectivity and selection in the sea scallop, *Placopecten magellanicus*, using RADseq-derived SNPs. Evolutionary Applications. 2017; 10:102–17. 10.1111/eva.12432 28035239 PMC5192885

[31] Levin PS, Möllmann C. Marine ecosystem regime shifts: Challenges and opportunities for ecosystem based management. Philosophical Transactions of the Royal Society B. 2015; 370:20130275.

[32] Mayr E. What Questions Does Ecology Ask? In: This is Biology: The science of the living world. Cambridge: Harvard University Press; 1997 pp. 207–226.

